# S-Thyroid Computer-Aided Diagnosis Ultrasound System of Thyroid Nodules: Correlation Between Transverse and Longitudinal Planes

**DOI:** 10.3389/fphys.2022.909277

**Published:** 2022-05-20

**Authors:** Keen Yang, Jing Chen, Huaiyu Wu, Hongtian Tian, Xiuqin Ye, Jinfeng Xu, Xunpeng Luo, Fajin Dong

**Affiliations:** ^1^ The Second Clinical Medical College, Jinan University, Shenzhen, China; ^2^ Department of Ultrasound, Shenzhen People’s Hospital (The Second Clinical Medical College, Jinan University; The First Affiliated Hospital, Southern University of Science and Technology), Shenzhen, China; ^3^ Department of Thyroid Surgery, Shenzhen People’s Hospital (The Second Clinical Medical College, Jinan University; The First Affiliated Hospital, Southern University of Science and Technology), Shenzhen, China

**Keywords:** computer-aided diagnosis, ultrasound, thyroid nodules, S-thyroid, transverse, longitudinal

## Abstract

**Introduction:** We compare the differences in the diagnostic results of S-thyroid, a computer-aided diagnosis (CAD) software, based on two mutually perpendicular planes.

**Methods:** Initially, 149 thyroid nodules confirmed by surgical pathology were enrolled in our study. CAD in our study was based on the ACR TI-RADS lexicon. *t* test, rank-sum test, and Chi-square test were used. The interclass correlation coefficient and Cohen’s kappa were used to explore the correlation between CAD features. Receiver operating characteristic was plotted for different combinations of CAD features.

**Results:** The patient’s age, transverse diameter, longitudinal diameter, shape, margin, echogenicity, echogenic foci, composition, TI-RADS classification, and risk probability of nodules in the transverse and longitudinal planes were related to thyroid cancer (*p* < 0.05). The AUC (95%CI) of TI-RADS classification in the transverse plane of CAD is better than that of the longitudinal plane [0.90 (0.84–0.95) vs. 0.83 (0.77–0.90), *p* = 0.04]. The AUC (95%CI) of risk probability of nodules in the transverse planes shows no difference from that in the longitudinal plane statistically [0.90 (0.85–0.95) vs. 0.88 (0.82–0.94), *p* = 0.52]. The AUC (95% CI), specificity, sensitivity, and accuracy [TI-RADS classification (transverse plane) + TI-RADS classification (longitudinal plane) + risk (transverse plane) + risk (longitudinal plane)] are 0.93 (0.89–0.97), 86.15%, 90.48%, and 88.59%, respectively.

**Conclusion:** The diagnosis of thyroid cancer in the CAD transverse plane was superior to that in the CAD longitudinal plane when using the TI-RADS classification, but there was no difference in the diagnosis between the two planes when using risk. However, the combination of CAD transverse and longitudinal planes had the best diagnostic ability.

## Introduction

Epidemiological studies show that thyroid cancer accounts for 3% of new cancers in women, with 32,130 cases compared to 12,150 cases in men. (Siegel et al., 2021). The increase in the number of thyroid cancer diagnoses is due in large part to the increasing use of diagnostic imaging technology and medical surveillance, as well as improved access to health care, all of which facilitate the detection of small, subclinical thyroid nodules and small thyroid cancers (Grani et al., 2020; Nambron et al., 2020).

Thyroid ultrasound (US) is the most effective tool for detecting thyroid lesions, especially when remnants of normal thyroid tissue are present, compared to other imaging studies such as computed tomography and magnetic resonance imaging (Grani and Fumarola, 2014; Hoang et al., 2018; Filetti et al., 2019). However, the repeatability and objectivity of the US are low, for the US highly dependent on operator experience and does not allow the analysis of image features quantitatively (Lee et al., 2016; Persichetti et al., 2020; Zhang et al., 2021).

To improve diagnostic accuracy, computer-aided diagnosis (CAD) systems have been developed (Shen et al., 2019; Zhang et al., 2021). CAD systems allow for quantitative assessment by efficiently analyzing large numbers of images, a computer-based approach that facilitates interpretation and diagnosis, and also reduces intra- and inter-observer variability (Singh et al., 2011). S-thyroid, similar to S-detect, is a computer-aided diagnostic software for ultrasound identification and differentiation of benign and malignant thyroid nodules. Some studies have investigated the diagnostic value of S-detect (Samsung Medison Co., Seoul, South Korea) for benign and malignant thyroid nodules (Choi et al., 2017; Kim et al., 2019; Xia et al., 2019; Barczyński et al., 2020; Wei et al., 2020), but no studies have been done on the diagnostic accuracy of S-thyroid for thyroid nodules. What is more, whether it is S-detect or S-thyroid, their diagnoses are based on a single ultrasound image. This is different from an ultrasonographer, who determines the benignity or malignancy of a thyroid nodule based on a combination of information from the transverse and longitudinal views of the thyroid nodule. However, no studies have yet examined the diagnostic variability of CAD between two mutually perpendicular views of the thyroid.

Consequently, the purpose of this study was to explore the differences between the diagnosis of thyroid nodules based on two mutually perpendicular planes of the S-thyroid software and the diagnostic efficacy of S-thyroid.

## Materials and Methods

### Informed Consent

This retrospective study was approved by the institutional ethics committee of our hospital.

### Patients

This retrospective study was approved by the appropriate institutional and research ethics committee. The inclusion and exclusion criteria are listed as follows:

1) US and CAD can detect the thyroid nodules of a patient.
2) Thyroid nodules range from 2 to 50 mm.
3) Thyroid nodule pathology was finally confirmed by surgical pathology.
4) Other non-thyroid cancers, such as lymphoma and metastatic cancers, were excluded.

**FIGURE 1:**
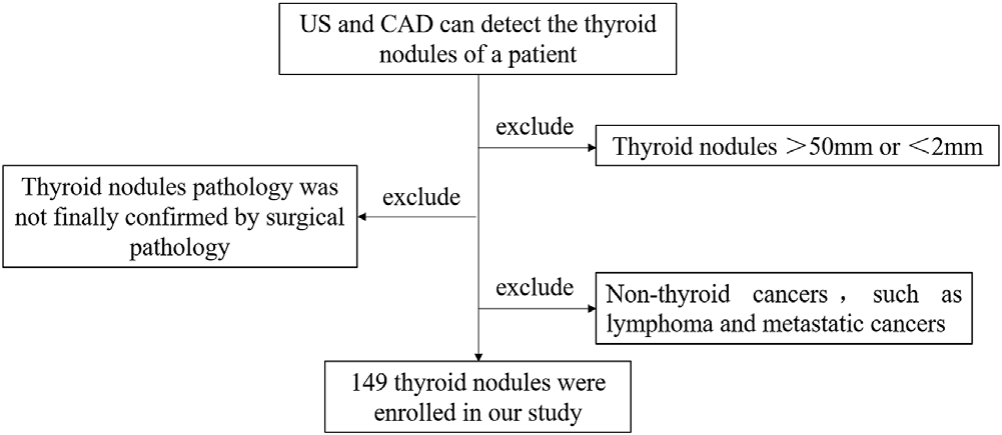
Flow chart.

The flow chart is shown in Figure 1.

## US and CAD Detection

The thyroid US detection was performed by an ultrasonographer with 15 years of experience in thyroid US detection using a 3–17 MHz linear array probe and a real-time US system (SonoScape Medical Corp., Shenzhen, Guangdong Province, China). First, the ultrasonographer performed a transverse scan to observe the entire thyroid gland, followed by a longitudinal scan. The transverse plane and longitudinal plane of the thyroid nodules with the most malignant signs have been preserved sequentially. Ultimately, the ultrasonographer measured the transverse diameter (TD), longitudinal diameter (LD), and anteroposterior diameter (AD) without knowing the pathology and CAD results.

CAD in our study was based on the American College of Radiology, Thyroid Imaging Reporting and Data System (ACR TI-RADS) lexicon (Tessler et al., 2018), and using S-thyroid software (SonoScape Medical Corp., Shenzhen, Guangdong Province, China). The thyroid nodules included in this study were not utilized for prior training or validation of the CAD system.

The CAD data in our study were obtained by the same ultrasonographer using the preserved images in both transverse and longitudinal planes. First, open S-thyroid, then without the need to outline the nodules manually, the CAD will automatically outline the thyroid nodule margin and display the following automatically as follows:

• TD, LD, AD;
• Composition: cystic or almost = 0; spongiform = 0; mixed cystic and solid = 1; solid or almost completely solid = 2;
• Echogenicity: anechoic = 0; hyperechoic or isoechoic = 1; hypoechoic = 2; very hypoechoic = 3;
• Shape: wider-than-tall = 0; taller-than-wide = 3;
• Margin: smooth = 0; ill-defined = 0; lobulated or irregular = 2; extra-thyroidal extension = 3;
• Echogenic Foci: none or large = 0; macrocalcifications = 1; peripheral (rim) calcifications = 2; punctate echogenic foci = 3;
• TI-RADS: TR1 = 0; TR2 = 2; TR3 = 3; TR4 = 4–6; TR5≥7.
• Risk: the CAD system assigns a score of 0–1, representing an increasing probability of malignancy in our study.

**FIGURE 2:**
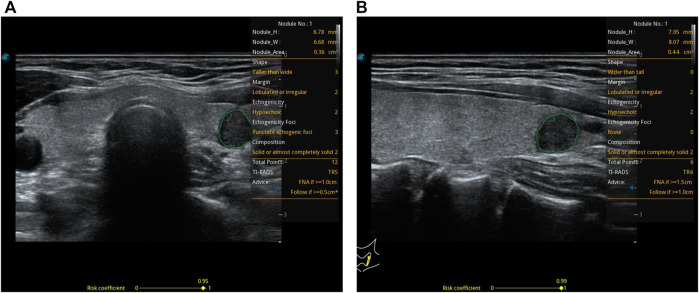
A 37-year-old woman with a thyroid nodule and pathological findings suggestive of a micro papillary thyroid carcinoma. S-thyroid analyzes the features of the lesion. **(A)** Transverse plane: shape = taller-than-wide; margin = lobulated or irregular; echogenicity = hypoechoic; echogenic foci = punctate echogenic foci; composition = solid or almost completely solid; TI-RADS classification = TR5; risk = 0.95. **(B)** Longitudinal plane: shape = wider-than-tall; margin = lobulated or irregular; echogenicity = hypoechoic; echogenic foci = none; composition = solid or almost completely solid; TI-RADS classification = TR4; risk = 0.99.

The relevant cases of CAD are shown in Figure 2.

What is more, two other sonographers with 5 and 10 years of experience in thyroid ultrasound detection, respectively, also read the saved images in the transverse and longitudinal planes and then graded the thyroid nodules according to ACR TI-RADS guidelines.

### Difference Between Transverse and Longitudinal Planes

To evaluate the difference between the transverse plane (view T) and longitudinal plane (view L), we calculated the plane difference (PD) and plane difference factor (PD^2^) through the equations given below:

$${\text{PD}}_{\text{composition}}{\;=\text{ composition}}_{\text{view L}}{\;-\text{composition}}_{\text{view T}}$$

$${\text{PD}}_{\text{echogenicity}}{=\text{echogenicity}}_{\text{view L}}{-\text{ echogenicity}}_{\text{view T}}$$

$${\text{PD}}_{\text{shape}}{=\text{shape}}_{\text{view L}}{\;-\text{shape}}_{\text{view T}}$$

$${\text{PD}}_{\text{margin}}{=\text{margin}}_{\text{view L}}{\;-\text{margin}}_{\text{view T}}$$

$${\text{PD}}_{\text{echogenic foci}}{=\text{echogenic foci}}_{\text{view L}}{\text{echogenic foci}}_{\text{view T}}$$

$${\text{PD}}_{\text{risk}}{=\text{ risk score}}_{\text{view L}}{-\text{ risk score}}_{\text{view T}}$$

$${\text{PD}}^{2}\;=\text{ PD ∗ PD}$$

### Clinical Findings

Patients’ gender, age, pathology results, and the location of the nodules were recorded. We divided the thyroid gland into three parts, including the left lobe, right lobe, and isthmus.

### Statistical Analysis

The statistical analysis was performed by R (https://www.r-project.org) and IBM SPSS 25, and the figures were assembled with Adobe Illustrator CS6 and GraphPad Prism 8. *t* test was used for the normally distributed numerical variables, the rank-sum test was used for the non-normally distributed numerical variables, and the Chi-square test was used for the disordered classification variables. *p* < 0.05, as standard, statistically significant variables were included for further study.

The result consistency of numerical variables between the transverse and longitudinal planes of CAD was analyzed by the interclass correlation coefficient (ICC) while the result consistency of disordered classification variables was analyzed by Cohen’s kappa. Multivariate logistic regression was used to construct the model, and then the ROC was plotted based on the results of multivariate logistic regression, to combine different CAD characteristics.

## Results

### Patients’ Sample

There were a total of 149 thyroid nodules (benign: malignant = 65:84) enrolled in our study. The median age of them was 44 years (interquartile range, 36–54 years). Among the 149 thyroid nodules, 69 nodules (46%) are located in the left lobe of the thyroid, 78 nodules (52%) are located in the right lobe of the thyroid, and 2 nodules (1%) are located in the isthmus of the thyroid. 25 (17%) of the patients were male and 124 (83%) of them were female. Gender (*p* = 0.53) and location (*p* = 1) were not statistically significant with thyroid cancer while age (*p* = 0.02) was statistically significant with thyroid cancer (Table1).

**TABLE 1:** Patients’ basic information.

Variable	Total (*n* = 149)	Benign (*n* = 65)	Malignant (*n* = 84)	*p*
Gender^c^				0.53
Male	25 (17)	9 (14)	16 (19)	
Female	124 (83)	56 (86)	68 (81)	
Age^d^	44 (36, 54	46 (41, 55	42 (34.75, 51.25	0.02
Location^c^				1
Left Lobe	69 (46)	30 (46)	39 (46)	
Right Lobe	78 (52)	34 (52)	44 (52)	
Isthmus	2 (1)	1 (2)	1 (1)	
Pathology				
Hashimoto thyroiditis		7 (11%)		
Nodular goiter		48 (74%)		
Follicular adenoma		8 (12%)		
Thyroid Hurthle cell adenoma		2 (3%)		
Papillary carcinoma			34 (40%)	
Micropapillary carcinoma			50 (60%)	
TI-RADS classification^a^ ^,^ ^d^				<0.01
1	19 (13)	17 (26)	2 (2)	
2	19 (13)	18 (28)	1 (1)	
3	10 (7)	8 (12)	2 (2)	
4	28 (19)	16 (25)	12 (14)	
5	73 (49)	6 (9)	67 (80)	
Risk^a^ ^,^ ^d^	0.75 (0.15, 0.95	0.14 (0.07, 0.37	0.94 (0.81, 0.97	<0.01
TI-RADS classification^b^ ^,^ ^c^				<0.01
1	17 (11)	16 (25)	1 (1)	
2	20 (13)	15 (23)	5 (6)	
3	11 (7)	8 (12)	3 (4)	
4	26 (17)	15 (23)	11 (13)	
5	75 (50)	11 (17)	64 (76)	
Risk^b^ ^,^ ^d^	0.87 (0.17, 0.97	0.21 (0.07, 0.57	0.96 (0.92, 0.98	<0.01

a Based on the CAD of the transverse plane.

b Based on the CAD of the longitudinal plane.

c Non-normally distributed numerical variables are shown by median (first quartile, third quantile).

d Disordered classification variables are shown by percentage.

### Characteristics of CAD

In the transverse plane of the thyroid, AD (*p* = 0.76) was not statistically significant with thyroid cancer while TD (*p* < 0.01), shape (*p* < 0.01), margin (*p* < 0.01), echogenicity (*p* < 0.01), echogenic foci (*p* < 0.01), composition (*p* < 0.01), TI-RADS classification (*p* < 0.01), and risk (*p* < 0.01) were statistically significant with thyroid cancer (Table1).

In the longitudinal plane of the thyroid, AD (*p* = 0.85) was not statistically significant with thyroid cancer while LD (*p* = 0.03), shape (*p* < 0.01), margin (*p* < 0.01), echogenicity (*p* < 0.01), echogenic foci (*p* < 0.01), composition (*p* < 0.01), TI-RADS classification (*p* < 0.01), and risk (*p* < 0.01) were statistically significant with thyroid cancer (Table1).

**FIGURE 3:**
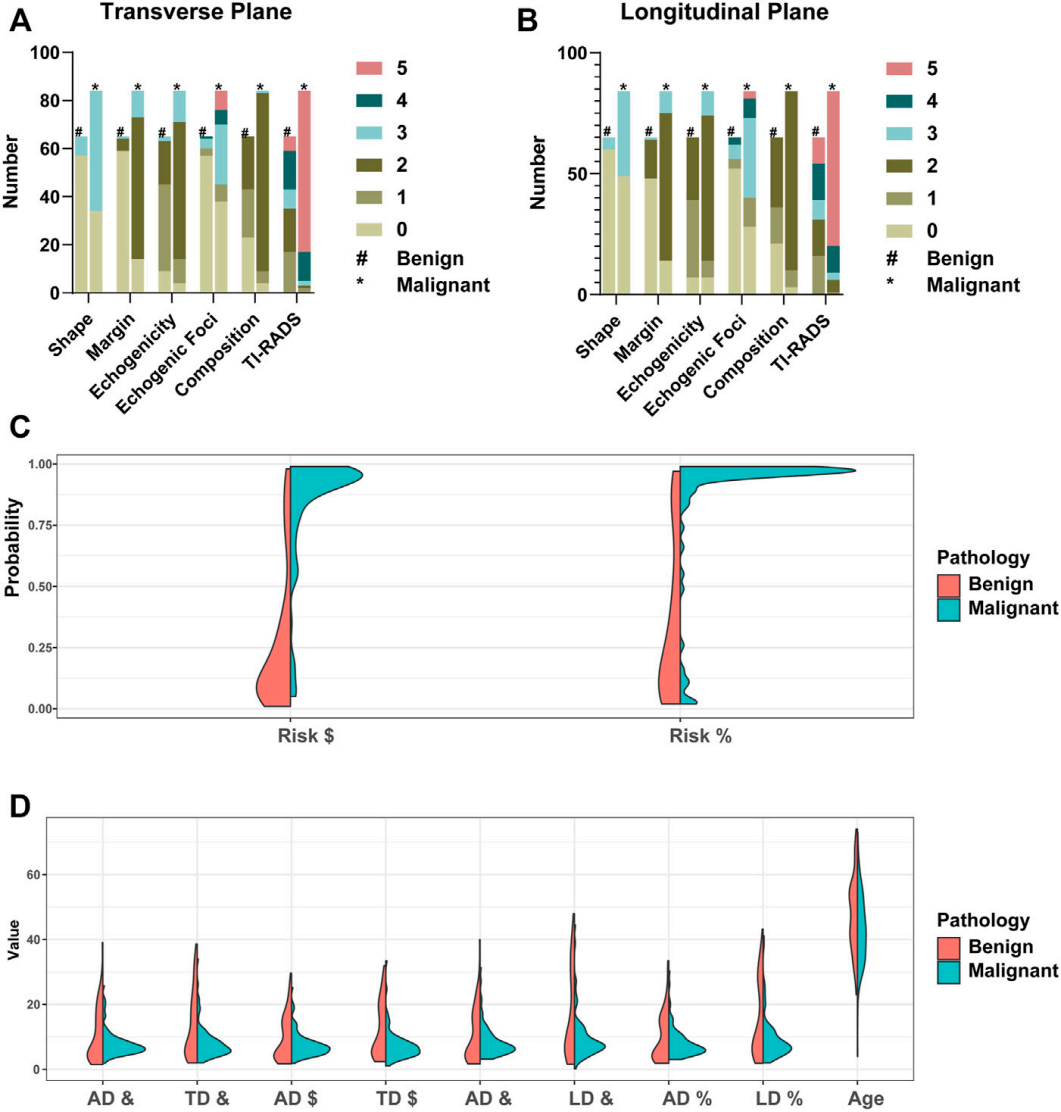
Patients’ data distribution between transverse and longitudinal planes. TI-RADS lexicon is shown in **(A,B)**. ^#^Benign group patients’ data distribution. *Malignant group patients’ data distribution. ^$^Based on the CAD of the transverse plane. ^%^Based on the CAD of the longitudinal plane. ^&^Based on the ultrasonographer’s diagnosis. TD: transverse diameter. LD: longitudinal diameter. AD: anteroposterior diameter. The risk score from the CAD system is shown in **(C)**. The TD, LD, AD, and age are shown in **(D)**.

Patients’ data distribution between transverse and longitudinal planes is shown in Figure 3.

### Difference Between Transverse and Longitudinal Planes

PD _risk_^2^ (*p* < 0.01), PD _shape_^2^ (*p* < 0.01), PD _echogenic foci_^2^(*p* < 0.01), and PD _composition_^2^(*p* < 0.01) were statistically significant with thyroid cancer (Table2). PD _risk_ (*p* = 0.66), PD _shape_ (*p* = 0.1), PD _margin_ (*p* = 0.05), PD _margin_^2^ (*p* = 0.35), PD _echogenicity_ (*p* = 0.21), PD _echogenicity_^2^ (*p* = 0.5), PD _echogenic foci_ (*p* = 0.55), and PD _composition_ (*p* = 0.11) were not statistically significant with thyroid cancer (Table2).

**TABLE 2:** Data distribution of PD and PD^2^.

Variable	Total (*n* = 149)	Benign (*n* = 65)	Malignant (*n* = 84)	*p*
PD _risk_	−0.02 (−0.12, 0.02)	−0.02 (−0.21, 0.04)	−0.02 (−0.06, 0.01)	0.66
PD _risk_^2^	0 (0, 0.04)	0.01 (0, 0.08)	0 (0, 0.01)	<0.01
PD _shape_	0 (0, 0)	0 (0, 0)	0 (0, 3)	0.1
PD _shape_^2^	0 (0, 9)	0 (0, 0)	0 (0, 9)	<0.01
PD _margin_	0 (0, 0)	0 (0, 0)	0 (0, 0)	0.05
PD _margin_^2^	0 (0, 1)	0 (0, 0)	0 (0, 1)	0.35
PD _echogenicity_	0 (0, 0)	0 (0, 0)	0 (0, 0)	0.21
PD _echogenicity_^2^	0 (0, 1)	0 (0, 1)	0 (0, 1)	0.5
PD _echogenic foci_	0 (0, 0)	0 (0, 0)	0 (0, 0)	0.55
PD _echogenic foci_^2^	0 (0, 1)	0 (0, 0)	0 (0, 4)	<0.01
PD _composition_	0 (0, 0)	0 (-1, 0)	0 (0, 0)	0.11
PD _composition_^2^	0 (0, 0)	0 (0, 1)	0 (0, 0)	<0.01

All the variables are shown by median (first quartile, third quantile), PD: plane difference, and PD^2^: plane difference factor.

### Consistency of CAD Features in Transverse and Longitudinal Planes

The ICC of AD, TD, and LD between the ultrasonographer’s diagnosis and CAD were 0.97 (0.95–0.98), 0.98 (0.98–0.99), and 0.98 (0.97–0.98), respectively, while the ICC of risk between the transverse plane and longitudinal plane of CAD was 0.81 (0.73–0.86), meaning that they have high consistency. The Kappa (mean ± standard error) of TI-RADS classification and shape between the transverse plane and longitudinal plane of CAD are 0.40 ± 0.05 and 0.34 ± 0.08, respectively, meaning that they have low consistency. The Kappa (mean ± standard error) of margin, echogenicity, echogenic foci, and composition between the transverse plane and longitudinal plane of CAD is 0.47 ± 0.06, 0.45 ± 0.06, 0.46 ± 0.06, and 0.54 ± 0.06, respectively, meaning that they have moderate consistency (Table 3).

**TABLE 3:** The consistency of CAD features.

	ICC (95% CI)^d^		Kappa^e^
Anteroposterior diameter^a^	0.97 (0.95–0.98)	Shape^c^	0.34 ± 0.08
Transverse diameter^a^	0.98 (0.98–0.99)	Margin^c^	0.47 ± 0.06
Anteroposterior diameter^b^	0.96 (0.94–0.97)	Echogenicity^c^	0.45 ± 0.06
Longitudinal diameter^b^	0.98 (0.97–0.98)	Echogenic foci^c^	0.46 ± 0.06
Risk^c^	0.81 (0.73–0.86)	Composition^c^	0.54 ± 0.06
TI-RADS classification^c^	0.40 ± 0.05		

a The consistency between the ultrasonographer’s diagnosis and CAD of the transverse plane.

b The consistency between the ultrasonographer’s diagnosis and CAD of the longitudinal plane.

c The consistency between the transverse plane and longitudinal plane of CAD.

d Shown by median (first quartile, third quantile).

e Shown by mean ± standard error.

CAD, computer-aided diagnosis; ICC, interclass correlation coefficient; CI: confidence interval.

### CAD Features’ Diagnosis Efficiency

**FIGURE 4:**
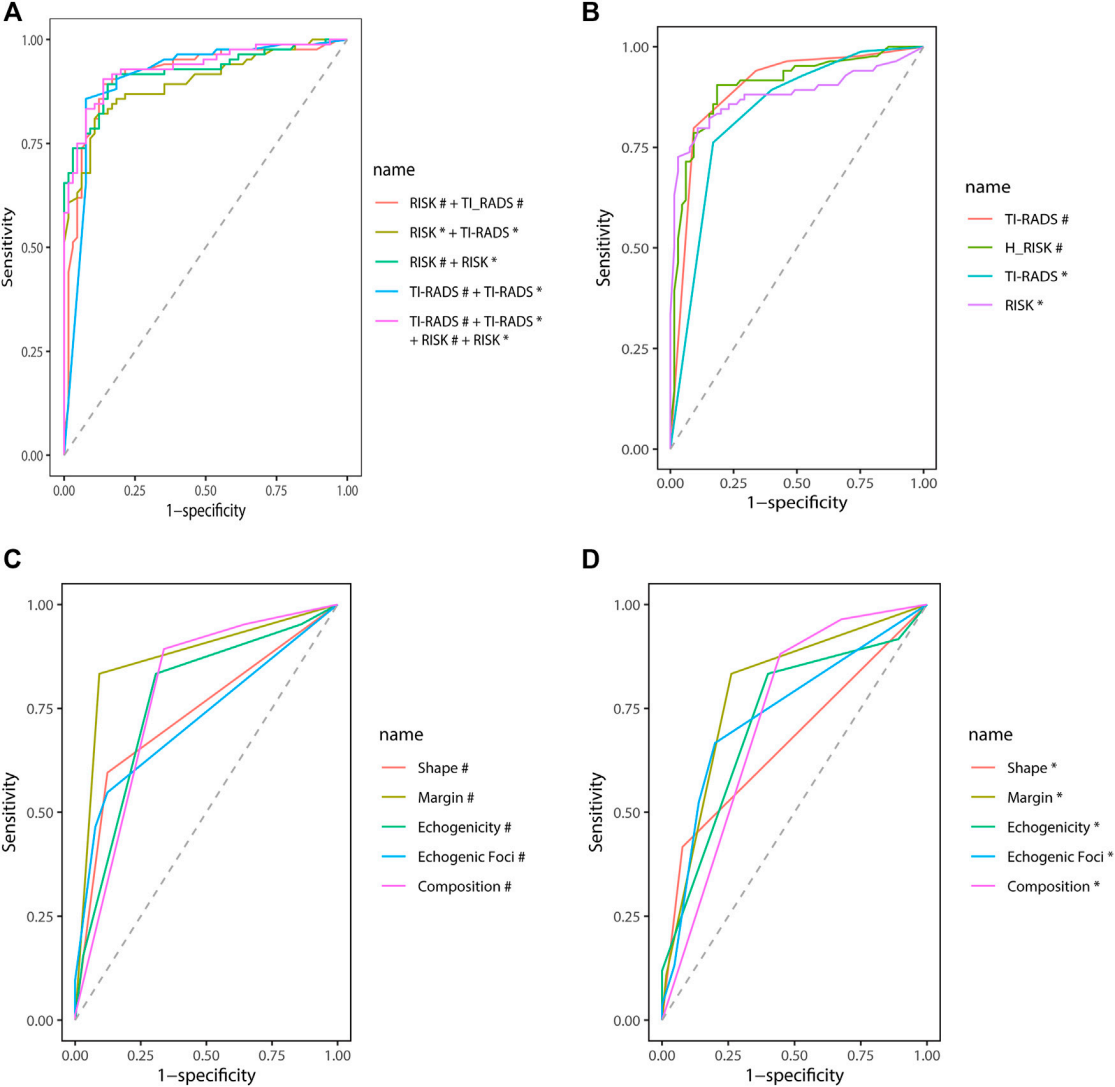
Receiver operating characteristic (ROC). ^#^Based on the CAD of the transverse plane. ^*^Based on the CAD of the longitudinal plane.

In two mutually perpendicular planes, Figure 4A and Table 4 demonstrate the ROC for various combinations of TI-RADS classifications and risk. TI-RADS classification and risk had the best diagnostic performance among the 7 features recorded by CAD in mutually perpendicular planes (Figures 4B–D).

**TABLE 4:** ROC of different combination of CAD features’ diagnosis efficiency.

	AUC (95%CI)	Threshold	Specificity	Sensitivity	Accuracy
TI-RADS^c^ + TI-RADS^d^ + risk^c^ + risk^d^	0.93 (0.89–0.97)	0.58	86.15	90.48	88.59
Risk^c^ + TI_RADS^c^	0.91 (0.86–0.96)	0.65	86.15	89.29	87.92
Risk^d^ + TI-RADS^d^	0.90 (0.85–0.95)	0.77	89.23	80.95	84.56
Risk^c^ + risk^d^	0.92 (0.88–0.97)	0.57	84.62	89.29	87.25
TI-RADS^c^ + TI-RADS^d^	0.91 (0.86–0.96)	0.66	92.31	85.71	88.59
TI-RADS^c^	0.90 (0.84–0.95)	4.5	90.77	79.76	84.56
Risk^c^	0.90 (0.85–0.95)	0.5	81.54	90.48	86.58
TI-RADS^d^	0.83 (0.77–0.90)	4.5	83.08	76.19	79.19
Risk^d^	0.88 (0.82–0.94)	0.94	96.92	72.62	83.22

a Based on the CAD of the transverse plane.

b Based on the CAD of the longitudinal plane.

c Non-normally distributed numerical variables are shown by median (first quartile, third quantile).

d Disordered classification variables are shown by percentage.

The AUC (95% CI) of TI-RADS classification in the transverse plane of CAD is better than that of the longitudinal plane [0.90 (0.84–0.95) vs. 0.83 (0.77–0.90), *p* = 0.04]. The AUC (95% CI) of risk in the transverse plane of CAD shows no difference from that in the longitudinal plane statistically [0.90 (0.85–0.95) vs. 0.88 (0.82–0.94), *p* = 0.52].

While combining the CAD features, the diagnosis efficiency will be better. The AUC (95% CI), specificity, sensitivity, and accuracy [TI-RADS classification (transverse plane) + TI-RADS classification (longitudinal plane) + risk (transverse plane) + risk (longitudinal plane)] are 0.93 (0.89–0.97), 86.15%, 90.48%, and 88.59%, respectively. The AUC (95% CI), specificity, sensitivity, and accuracy [TI-RADS classification (transverse plane) + risk (transverse plane)] are 0.91 (0.86–0.96), 86.15%, 89.29%, and 87.92%, respectively. The AUC (95% CI), specificity, sensitivity, and accuracy [TI-RADS classification (longitudinal plane) + risk (longitudinal plane)] are 0.90 (0.85–0.95), 89.23%, 80.95%, and 84.56%, respectively. The AUC (95%CI), specificity, sensitivity, and accuracy [risk (transverse plane) + risk (longitudinal plane)] are 0.92 (0.88–0.97), 84.62%, 89.29%, and 87.25%, respectively. The AUC (95% CI), specificity, sensitivity, and accuracy [TI-RADS classification (transverse plane) + TI-RADS classification (longitudinal plane)] are 0.91 (0.86–0.96), 92.31%, 85.71%, and 88.59%, respectively.

Of the diagnoses made by ultrasonographers of different seniority, the AUC (95% CI), specificity, sensitivity, and accuracy of the 15 years experienced ultrasonographer were 0.90 (0.85–0.95), 81.54%, 89.29%, and 85.91% and that of 10 years experienced ultrasonographer were 0.88 (0.82–0.94), 86.15%, 83.33%, and 84.56% and that of 5 years experienced ultrasonographer were 0.86 (0.80–0.92), 84.62%, 79.76%, and 81.88%, respectively.

## Discussion

The incidence of thyroid cancer is increasing year by year. However, the mortality rate of thyroid cancer has not changed (Sosa et al., 2013; Ahn et al., 2016; Leboulleux et al., 2016; Vaccarella et al., 2016). Thus, it is necessary for clinicians to reduce punctures and surgeries for thyroid nodules. ACR TI-RADS, a lexicon for imaging practitioners reporting thyroid nodules, has developed a standardized risk stratification system for thyroid nodules (Tessler et al., 2017; Tessler et al., 2018). Unlike ultrasonographers who are rated according to ACR TI-RADS, S-thyroid is reproducible and objective according to ACR TI-RADS. The objective of our study is to inform clinicians on how to respond when CAD scores different risk scores based on two mutually perpendicular planes so that better clinical protocol decisions can be made that are more beneficial to patients.

We can see that TD, LD, and shape were statistically significant with thyroid cancer while AD was not, and the TD and LD of malignant nodules are smaller than those of benign nodules. Since benign and cystic nodules have softer nodules and less infiltration of surrounding tissue and are therefore more easily compressed than malignant nodules (Yoon et al., 2010), 56%–89% of papillary thyroid cancers showed dense fibrosis (Vickery, 1983; Isarangkul, 1993). This may be the reason why malignant nodules are less likely to be compressed. However, the reasons for the result of our study are more likely due to selection bias. In the case of small nodules, it is likely that only those with suspicious features underwent a biopsy or surgery. Further expansion of the sample is needed to compare the statistical results again.

In our study, we observed that the ACR TI-RADS lexicon diagnosis of CAD based on the transverse plane did differ from that of CAD based on the longitudinal plane, but the difference was not statistically significant with thyroid cancer, while the square of the difference was statistically significant with thyroid. Surprisingly, the correlation of the TI-RADS classification of CAD based on two mutually perpendicular planes was low, but the correlation of risk was high. Therefore, the ultrasonographer or clinician should give priority to the risk score of CAD over the TI-RADS classification of CAD when interpreting CAD reports. Similarly, the correlations of margin, echogenicity, echogenic foci, and composition based on two mutually perpendicular planes of CAD were moderate. This may be the reason for the low correlation of the TI-RADS classification based on two mutually perpendicular planes in our study. We know that the images of thyroid nodules are not consistent in both planes, so ultrasonographers base their grading of thyroid nodules on the combined scan. This is different from the TI-RADS classification of CAD, which is why the previous results occur. However, this phenomenon has not been analyzed before, so what should an ultrasonographer or clinician do when interpreting CAD results that are inconsistent based on two mutually perpendicular planes?

Therefore, this study also investigated the diagnostic efficacy of TI-RADS classification and risk in CAD transverse and longitudinal planes. It was found that the diagnosis of thyroid cancer in the CAD transverse plane was superior to the CAD longitudinal plane when using the TI-RADS classification, but there was no difference in the diagnosis between the two planes when using risk. What is more, the combination of both planes can improve the diagnosis of thyroid cancer. Therefore, the ultrasonographer or clinician should not interpret the CAD results based on one plane alone but should combine the results of both the transverse and longitudinal planes.

Wei Q et al. found that S-detect can improve diagnostic performance for less experienced radiologists, and the sensitivity and specificity of S-detect are 91.3% and 65.2%, respectively (Wei et al., 2020). Kim HL et al. evaluated the diagnostic performance of S-Detect 1 and S-Detect 2 for detecting thyroid cancers and found that the sensitivity and specificity of S-Detect 1 are 80.2% and 82.6% and that of S-Detect 2 are 81.4% and 68.2%, respectively (Kim et al., 2019). Xia S et al. found that CAD presents a higher sensitivity but lower specificity in identifying malignant thyroid nodules compared to experienced radiologists, and the sensitivity and specificity of S-detect are 90.5% and 41.2%, respectively (Xia et al., 2019). Barczyński M et al. found that the CAD system has similar sensitivity to classify thyroid lesions as a surgeon with expert US skills (Barczyński et al., 2020). In conclusion, all the aforementioned studies demonstrated the high sensitivity and low specificity of S-detect in the diagnosis of malignant thyroid nodules. However, in our study, S-thyroid showed high sensitivity and specificity for identifying thyroid cancer with a combination of transverse and longitudinal planes, 90.48% and 86.15%, respectively. Interestingly, S-thyroid results in either transverse or longitudinal planes alone have low sensitivity and high specificity in the identification of thyroid cancer. The reason for this result may be due to the different algorithms of S-thyroid and S-detect, or it may be due to the fact that the study sample of this study is different from the study sample of the previous study, and further comparison of the two CAD software with the same patient sample is needed.

There are several limitations to our study. First, we selected patients who had undergone surgery, so the ratio of benign to malignant thyroid nodules was not correct, and there were more malignant nodules than benign ones, which may affect the diagnostic performance of the CAD system. Second, non-mass lesions were not included in the study population because the CAD analysis was limited to non-mass lesions. Last but not the least, the number of patient cases in this study was too small, and further sample size studies are needed.

## Conclusion

In our study, we explored the diagnostic capability of S-thyroid, using CAD software for thyroid nodules based on two mutually perpendicular planes and found that the best diagnostic capability was achieved with a combination of CAD transverse and longitudinal planes.

## Data Availability

The original contributions presented in the study are included in the article/Supplementary Material, further inquiries can be directed to the corresponding authors.

## Abbreviations

US, ultrasound; CAD, computer-aided diagnosis; PD, plane difference; PD2, plane difference factor; ROC, receiver operating characteristic; AUC, area under ROC curve; TD, transverse diameter, LD, longitudinal diameter; AD, anteroposterior diameter; ICC, interclass correlation coefficient; TI-RADS, Thyroid Imaging Reporting and Data System; and ACR TI-RADS, American College of Radiology, Thyroid Imaging Reporting and Data System.
